# Clathrin-dependent endocytosis is associated with RNAi response in the western corn rootworm, *Diabrotica virgifera virgifera* LeConte

**DOI:** 10.1371/journal.pone.0201849

**Published:** 2018-08-09

**Authors:** Daniele H. Pinheiro, Ana M. Vélez, Elane Fishilevich, Haichuan Wang, Newton P. Carneiro, Arnubio Valencia-Jiménez, Fernando H. Valicente, Kenneth E. Narva, Blair D. Siegfried

**Affiliations:** 1 Entomology and Nematology Department, Charles Steinmetz Hall, University of Florida, Gainesville, Florida, United States of America; 2 Department of Chemistry, Federal University of Lavras, Lavras, Minas Gerais, Brazil; 3 Department of Entomology, Entomology Hall, University of Lincoln, Lincoln, Nebraska, United States of America; 4 Corteva Agriscience™, Agriculture Division of DowDuPont™, Indianapolis, Indiana, United States of America; 5 Nucleus of Applied Biology, Embrapa Maize and Sorghum, Sete Lagoas, Minas Gerais, Brazil; 6 Departamento de Producción Agropecuaria, Universidad de Caldas, Manizales, Caldas, Colombia; University of Tennessee, UNITED STATES

## Abstract

The cellular uptake of dsRNA after dietary exposure is critical for RNAi efficiency; however, the mechanism of its uptake in many insects remains to be understood. In this study, we evaluated the roles of the endocytic pathway genes *Clathrin heavy chain* (*Chc*), *Clathrin adaptor protein AP50*, *ADP ribosylation factor-like 1* (*Arf72A*), *Vacuolar H*^*+*^ *ATPase 16 kDa subunit* (*Vha16*), and *small GTPase Rab7* and putative *sid*-*1*-like genes (*silA* and *silC*) in RNAi response in western corn rootworm (WCR) using a two-stage dsRNA exposure bioassay. Silencing of *Chc*, *Vha16*, and *AP50* led to a significant decrease in the effects of *laccase2* dsRNA reporter, indicating that these genes are involved in RNAi response. However, the knockdown of either *Arf72A* or *Rab7* did not suppress the response to *laccase2* dsRNA. The silencing of the *silC* gene did not lead to a significant reduction in mortality or increase in the expression of *V-ATPase A* reporter. While the silencing of the *silA* gene significantly decreased insect mortality, significant changes in *V-ATPase A* expression were not detected. These results suggest that clathrin-dependent endocytosis is a biological mechanism that plays an important role during RNAi response in WCR adults. The fact that no definitive support for the roles of *silA* or *silC* in RNAi response was obtained support the idea that RNAi response varies greatly in different insect species, demanding additional studies focused on elucidating their involvement in this mechanism.

## Introduction

Experiments conducted in several insect orders have shown that RNAi can be used as a potential tool for insect pest management by induction of an RNAi response after ingestion of dsRNA [1–7]. The mechanism that allows uptake of the dsRNA from the gut lumen by midgut cells and the subsequent systemic spread from cell to cell is likely to have significant influence on the efficiency of RNAi. Considering that the level of RNAi response across insect orders is highly variable [8–10], a better understanding of this process could aid in the development and improvement of RNAi-based technologies for insect pest management.

The western corn rootworm (WCR), *Diabrotica virgifera virgifera* LeConte, is one of the most important insect pests of maize throughout the U.S. Corn Belt [11]. Control failures of WCR with synthetic insecticides, crop rotation, and transgenic plants expressing *Bacillus thuringiensis* toxins have become a serious problem and new management strategies are urgently needed [12–18]. To complement current management strategies, transgenic maize events based on RNAi for WCR control are likely to be deployed by the end of the decade [7].

WCR exhibits a robust systemic RNAi response induced by direct injection of dsRNA [19], feeding of dsRNA provided in artificial diet [20–22], or transgenic plants expressing dsRNA [2, 20]. Understanding the mechanism of dsRNA uptake and the spread of the RNAi signal will contribute not only to the improvement of RNAi efficiency for the management of WCR and other insect pests but will also generate valuable information about potential and some specific mechanisms of insect resistance to RNAi-based technologies.

The systemic RNA interference-defective-1 (SID-1) protein is perhaps the best studied factor required for dsRNA uptake and systemic RNAi [23, 24]. In *Caenorhabditis elegans*, the SID-1 multi-transmembrane domain protein is present in the cell membrane and functions as a channel for the passive transport of dsRNA between cells [23]. In addition to SID-1, other proteins are involved in dsRNA uptake in *C*. *elegans*, including SID-2, SID-3, and SID-5. SID-2 is responsible for the active import of environmental dsRNA from the intestinal lumen [25, 26], while SID-3 is a conserved tyrosine kinase that allows the entry of dsRNA into the cells [27]. In contrast, SID-5 is an endosome-associated protein, believed to be involved in the release of dsRNA from the endosome and movement of the RNAi signal from cell to cell [28, 29].

Homologous sequences of the *sid-1* gene have been identified in many species of different insect orders, except for Diptera [30]. In WCR, only two *sid-1-*like genes have been found and the phylogenetic analysis of the deduced proteins indicates that they are orthologous to the *Tribolium silA* and *silC* genes [31]. An association between the presence of *sid-1-*like genes and the systemic spread of dsRNA was initially proposed since *sid-1*-like genes are absent in *Drosophila melanogaster* and other dipterans, species that do not display a robust systemic RNAi response. However, subsequent studies on other insect orders demonstrated that the presence of *sid-1-*like genes does not necessarily result in a robust systemic RNAi response. For example, the silkworm, *Bombyx mori*, harbors three *sid-1-*like genes, but it does not exhibit a substantial systemic RNAi response [30].

Endocytosis has been suggested as an alternative dsRNA uptake mechanism in *D*. *melanogaster* S2 cells [32, 33]. The silencing of the *Chc* gene, which plays a critical role in clathrin-dependent endocytosis, reduced the lethality of *D*. *melanogaster* S2 cells after exposure to *Ubiquitin* (*Ubi-p63E*) dsRNA, an essential gene for cell viability [33]. A functional screening of a dsRNA library from *D*. *melanogaster* S2 cells found several genes involved in the endocytic pathway [32], including *Clathrin heavy chain* (*Chc*), *Clathrin adaptor protein AP50*, that is also known as the μ *subunit* of the AP2 adapter complex (*AP-2*μ*/AP50*) [34], *ADP ribosylation factor-like 1* (*Arl1/Arf72A*) [35], *Vacuolar H*^*+*^ *ATPase* (*V-ATPase*) 16 kDa (CG3161) and SFD (CG17332) subunits, and small GTPase *Rab7* to be necessary for cellular uptake of dsRNA [32]. The proteins encoded by these genes have been associated with various functions in the clathrin-dependent endocytosis. Chc is the major component of clathrin-coated pits formed on the inner surface of the cytoplasmic membrane [34]; AP50 acts linking the clathrin to its cargo [34] and Arl1/Arf72A is involved in endosomal trafficking at trans-Golgi [36, 37], while Vacuolar H^+^ ATPase (Vha16) and Rab7 play critical roles in endosome maturation by acidifying endosomes and promoting late endosome fusion, respectively [32, 38].

The requirement of the endocytic pathway for cell entry of dsRNA has also been suggested in other insects including the desert locust, *Schistocerca gregaria* [39], the oriental fruit fly, *Bactrocera dorsalis* [40], the red flour beetle, *Tribolium castaneum* [41], and Colorado potato beetle, *Leptinotarsa decemlineata* [42, 43]. The silencing of different genes directly related to clathrin-dependent endocytosis, including, *Chc*, *Arf72A*, *Rab7*, *AP50*, and *Vacuolar H*^*+*^ *ATPase SFD subunit* (*VhaSFD*) was able to block significantly the RNAi response in *T*. *castaneum*, supporting the involvement of endocytosis in dsRNA uptake [41]. Similarly, the knockdown of *Chc* and *Vacuolar H*^*+*^ *ATPase 16 kDa subunit* (*Vha16*) in *S*. *gregaria* also diminished the RNAi response in this insect [39].

While it is possible that both clathrin-dependent endocytosis and the SIL proteins are involved in the RNAi effect and the uptake/spread of the dsRNA in WCR, a direct comparison of their impact on RNAi response will help to identify key genes involved in these processes and the reasons for a potential diverse uptake/spread response during RNAi mechanism. By testing both endocytic genes and *sil* genes in *L*. *decemlineata* [42], it was established that endocytosis is the major contributor to successful RNAi in this insect. Since WCR is a major agricultural pest that is readily susceptible to RNAi and is a robust laboratory model, elucidating key pathways involved in its RNAi response can pave the way to understanding of environmental RNAi in other insects.

In the present study, we report that interruption of the endocytic pathway by silencing *AP50*, *Chc*, and *Vha16* genes decreased the suppression of the non-lethal reporter gene, *laccase2*, demonstrating that clathrin-dependent endocytosis is involved in RNAi response in WCR adults. In contrast, the knockdown of *sil* genes yielded mixed results. *silA* knockdown generated a reduced RNAi response in WCR adults exposed to dsRNA of the lethal reporter gene, *V-ATPase A*, that could not be supported at the transcript level. The knockdown of *silC* or both *sil* genes simultaneously did not affect the RNAi response. These results do not provide a strong evidence for the involvement of *sil* genes in the RNAi response in WCR, yet they do not rule it out.

## Material and methods

### Insects and diet

Newly emerged non-diapausing WCR adults were purchased from Crop Characteristics Inc. (Farmington, MN). The artificial diet used in all bioassays was modified from Branson and Jackson [44]. The diet consisted of 6 grams of the dry ingredients reported by Branson and Jackson [44], 12.5 ml of water, 0.365 g of agar, 0.7 ml of glycerol, and 27.5 μl of a solution of 47% propionic acid and 6% phosphoric acid to reduce microbial contamination. The diet was dispensed into a Petri dish, allowed to solidify at room temperature, and diet plugs were cut using a cork borer (4 mm diameter). WCR adults were kept in a growth chamber at 23 ± 1°C, 75 ± 5% relative humidity with 16:8 photoperiod.

### Identification of endocytic pathway genes and putative *sid-1*-like in the WCR transcriptome

Nucleotide sequences encoding *Clathrin heavy chain* (*Chc*) (KJ135005.1) and *Vacuolar H*^*+*^ *ATPase 16kDa subunit* (*Vha16*) (KJ135006.1) from *S*. *gregaria* were used as query sequences to search for putative homologs in the WCR transcriptome. Putative *sid-1-*like (*silA* and *silC*) and clathrin-mediated endocytosis genes were identified by BLAST searching the WCR transcriptome database described by Eyun et al. [45], using the reported sequences from *S*. *gregaria* and *T*. *castaneum* [30, 39, 41]. The sequences encoding *Clathrin adaptor protein AP50* (*AP50*) (KJ476827), *small GTPase Rab7* (*Rab7*) (KJ476829), *ADP-ribosylation factor-like protein 1* (*Arf72A*) (XM_967932.3), *systemic RNA interference defective-1-related A* (*Sil-1A*) (NM_001105542.1), and *systemic RNA interference defective-1-related C* (*Sil-1C*) (NM_001105658.1) from *T*. *castaneum* were used as queries. The identified putative WCR orthologs [*Chc* (KX965603), *Vha16* (KX965604), *AP50* (KX965605), *Arf72A* (KX965607), *Rab7* (KX965606), *silA* (KX965608) and *silC* (KX965609)] were deposited in GenBank.

### Double stranded RNA (dsRNA) preparation

Total RNA was isolated from the whole body of a single WCR adult using GeneJET RNA Purification Kit (Fermentas-Thermo Scientific, Waltham, MA) following manufacturer’s instructions. First-strand cDNA was generated with 500 ng of total RNA using the QuantiTect Reverse Transcription Kit (Qiagen, Valencia, CA). Gene-specific primers were designed with Primer3Plus software [46, 47] to amplify *silA*, *silC*, *Chc*, *Vha16*, *AP50*, *Arf72A*, *Rab7*, *laccase2*, and *GFP* gene. All primers included the T7 promoter sequence at the 5’ end. The primers described by Rangasamy and Siegfried [22] were used for the amplification of *V-ATPase A* gene (S1 Table). All PCR amplification products were sequenced to confirm the identity and specificity. For negative control, the non-specific green fluorescence protein gene (*GFP*) was amplified from the pIZT/V5-His expression vector (Invitrogen, USA).

The amplified PCR products were purified using QIAquick PCR Purification Kit (Qiagen, Valencia, CA) and used as templates for *in vitro* dsRNA synthesis using the MEGAscript high-yield transcription Kit (Applied Biosystems Inc., Foster City, CA). The synthesized dsRNAs were purified using the RNeasy Mini Kit (Qiagen, Valencia, CA) according to the manufacturer’s instructions, quantified using NanoDrop ND-1000 spectrophotometer (Thermo Scientific, Franklin, MA), and then examined by agarose gel electrophoresis to determine their purity and integrity.

### Insect bioassay for functional analysis of endocytic genes

To determine the involvement of the clathrin-dependent endocytosis in dsRNA uptake in WCR, we used a similar approach to that described by Velez et al. [48]. However, we modified the time between the first and second dsRNA exposure to account for time necessary to generate gene silencing and the effect of the gene silencing on the survival of WCR adults. The bioassays with *Chc* and *Vha16* were performed in five days since silencing of these genes affected the survival of insects, with the first and second dsRNA exposure on day zero and day three.

**Fig 1:**
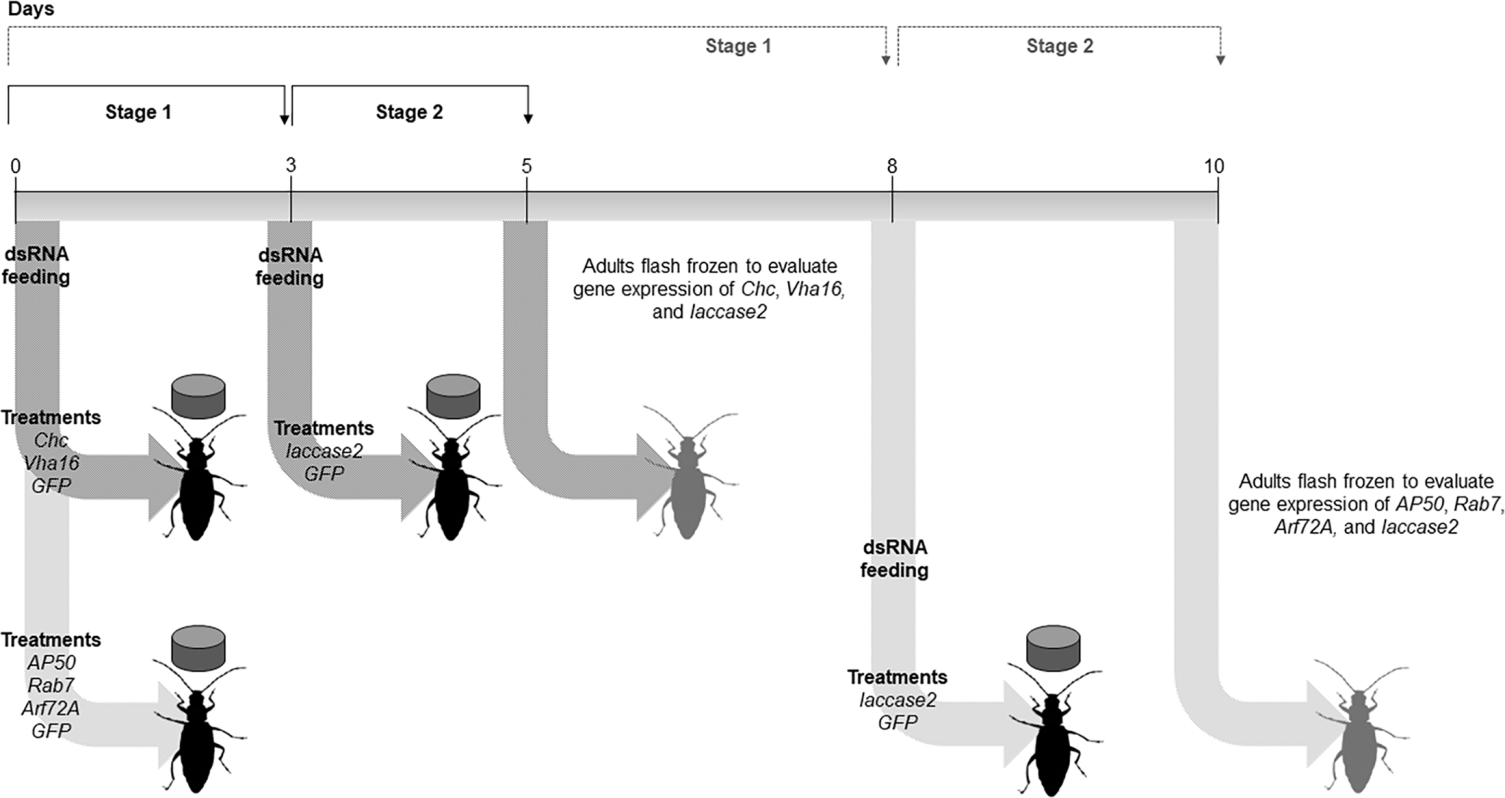
Two-step bioassay to determine the effect of the suppression of the endocytic genes on the expression of *laccase2* in WCR adults.

The bioassays with *AP50*, *Arf72A*, and *Rab7* were performed over ten days since these genes had weak knockdown after three days of exposure to dsRNA. The first and second dsRNA exposures occurred on days zero and eight (Fig 1). In this experiment, both dsRNA exposures were performed by feeding. During the first dsRNA exposure, WCR adults were fed with diet plugs treated with 600 ng of *Chc*, *Vha16*, *AP50*, *Arf72A*, *Rab7*, or *GFP* dsRNA every other day. For the second dsRNA exposure, new diet plugs coated with 600 ng of *laccase2* or *GFP* dsRNA were provided to WCR adults on day three or eight, depending on the group treatment. One WCR adult was collected per replication from all the treatments immediately before and two days after the second dsRNA exposure, flash-frozen in liquid nitrogen, and stored at -80°C for total RNA extraction. Each assay was performed in triplicate with a total of 14 beetles per treatment.

### Insect bioassay for functional analysis of *silA* and *silC* genes

To determine if the lethal effect of the *V-ATPase A* dsRNA would be altered by the knockdown of *sil* genes we used a similar approach to that described by Velez et al. [48] with slight modifications. Briefly, a volume of 0.6 μl of *silA* and *silC* dsRNA at 1 μg/μl, and a mixture of *silA* and *silC* dsRNA containing equal amounts of both dsRNAs, for a total of 600 ng, were injected into individual beetles. Insects were injected between the coxae of the last pair of legs using a glass capillary syringe and then fed with untreated diet.

To determine if competitive inhibition of dsRNA occurred when multiple species-specific dsRNAs were provided to beetles, WCR adults were also injected with dsRNA targeting the non-lethal gene *laccase2*, which is required for cuticular pigmentation [49]. WCR adults injected with *GFP* dsRNA were used as controls and un-injected adults were used to evaluate the mortality associated with the injury caused by the injection. The beetles were anesthetized with carbon dioxide to facilitate injections.

**Fig 2:**
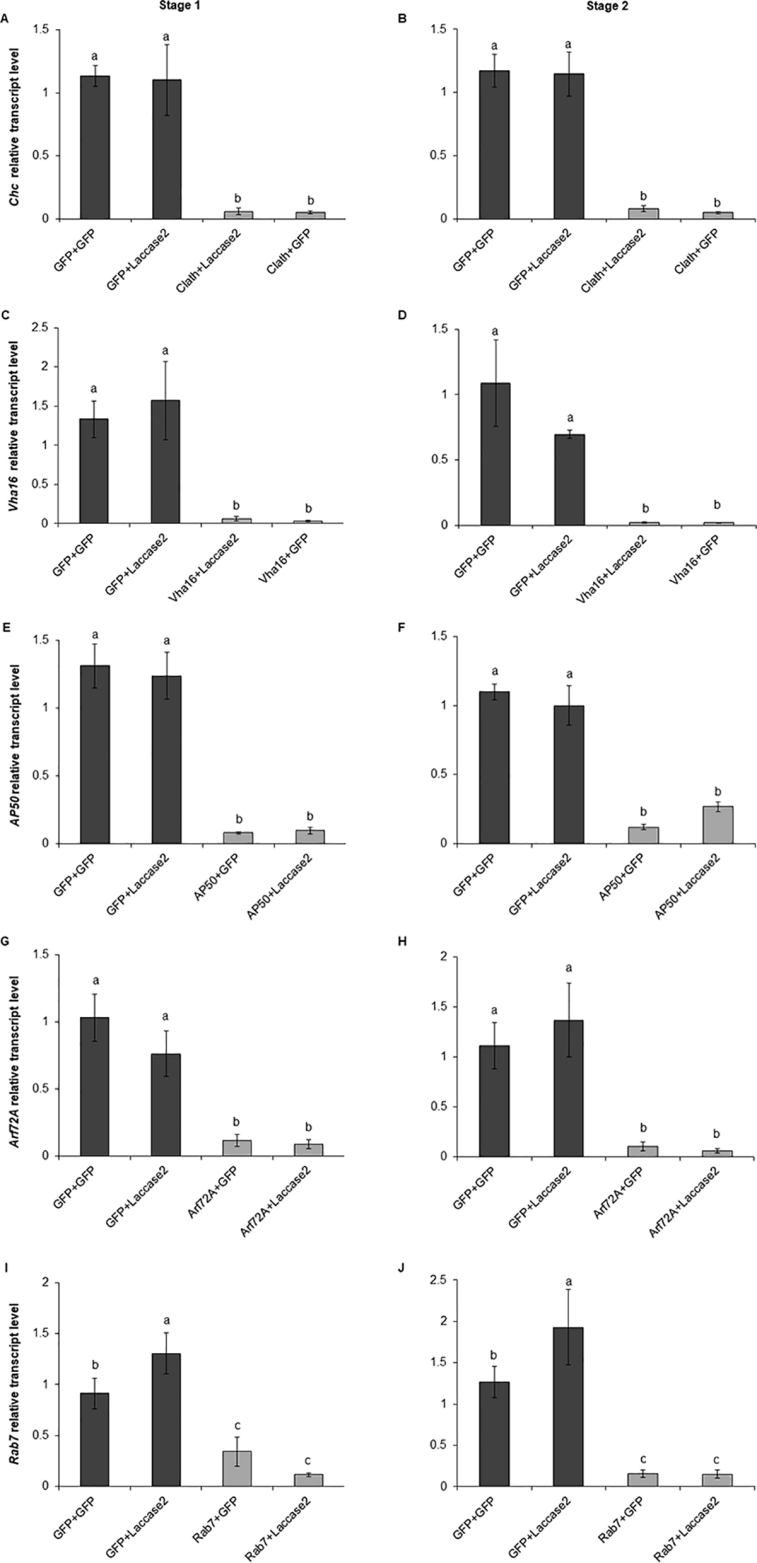
Relative transcript levels of the endocytosis-related genes after dsRNA exposure. Relative transcript levels of *Chc* (A and B), *Vha16* (C and D), *AP50* (E and F), *Arf72A* (G and H), and *Rab7* (I and J) genes in WCR adults after the first (stage 1) and second (stage 2) dsRNA exposure evaluated by RT-qPCR. Values shown are the means and standard errors (±SE) of three biological replicates each with two technical replicates. Different letters represent significant differences at *p*-value < 0.05.

For the secondary exposure to dsRNA, diet plugs (4 mm diameter x 2 mm height) were surface-treated with 500 ng of *V-ATPase A* dsRNA and provided to beetles three, five, seven, nine, and eleven days after injection. For the remainder of the assays, beetles were provided with untreated diet for a total of 14 days (Fig 2). On day seven, one beetle per replication was collected from all the treatments, flash frozen in liquid nitrogen and stored at -80°C for total RNA extraction. A total of 16 beetles were used in each treatment and mortality was recorded daily until day 14. Each assay was performed in triplicate.

### Quantitative Real Time PCR (RT-qPCR)

Total RNA was isolated from the whole bodies of adults using GeneJET RNA Purification Kit (Fermentas-Thermo Scientific, Waltham, MA) following the manufacturer’s recommendations. cDNA was synthetized with the QuantiTect Reverse Transcription Kit (Qiagen, Valencia, CA) using 500 ng of RNA, following the manufacturer’s instructions. The RT-qPCR reactions included 1 μl of cDNA diluted 50X, 5 μl of Fast SYBR^®^ Green Master Mix (Applied Biosystems, Foster City, CA), 0.2 μl at 10 μM of each primer, and 3.6 μl of nuclease-free water, for a total volume of 10 μl. The primers were designed with Primer3Plus [46, 47] and validated by PCR amplification efficiencies (E) and correlation coefficients (R^2^) analysis (S2 Table).

Both primer efficiency test and RT-qPCR were performed on a 7500 Fast RT-PCR System (Applied Biosystems, Grand Island, NY). The thermocycler conditions were one cycle at 95°C for 20 s, followed by 40 cycles of denaturation at 95°C for 3 s and annealing/extension at 60°C for 30 s. At the end of each PCR reaction, a melting curve was generated to confirm a single peak and rule out the possibility of primer-dimers and nonspecific product formation. The expression of the genes was calculated using the 2^-ΔΔCT^ method [50]; using *actin* as reference gene which has previously demonstrated to be stable under experimental conditions in which WCR were exposed to dsRNA treatments [51]. RT-qPCR analysis was performed with three biological replicates and two technical replicates.

### Statistical analysis

Gene expression and mortality were subjected to an analysis of variance (ANOVA) using the PROC GLIMMIX procedure; least-squares differences were used to perform pairwise comparisons between treatments. All statistical analyses were performed using SAS Software version 9.4 [52]. Data were expressed as mean ± standard error of the mean (SE), and values of *p* < 0.05 were considered statistically significant.

## Results

### Identification of putative clathrin-dependent endocytic pathway and *sid-1*-like genes in the WCR transcriptome

A BLAST search allowed the identification of putative *Chc* (NCBI ID: KX965603), *Vha16* (KX965604), *AP50* (KX965605), *Arf72A* (KX965607), *Rab7* (KX965606), *silA* (KX965608) and *silC* (KX965609) genes in the WCR transcriptome database. We also searched for the putative *silB* gene, however, no orthologs to the *Tribolium silB* gene were found in the WCR transcriptome. Additional BLASTX analysis revealed that the deduced WCR protein sequences display high sequence identity to their orthologs from different insect species and shared the highest identity with proteins from *T*. *castaneum* or *L*. *decemlineata* [above 67%] (S3 Table). A reverse BLAST of the *C*. *elegans* proteome (http://www.wormbase.org/) using the putative WCR SILA and SILC proteins has revealed CHolesterol UPtake associated protein CHUP-1 as the closest homolog in *C*. *elegans* (S1 Fig).

### Knockdown of clathrin-dependent endocytosis elements impedes WCR’s RNAi response

To examine the hypothesis that the clathrin-dependent endocytosis is involved in RNAi response in WCR adults, we used an “RNAi-of-RNAi” approach (Fig 1), which has been successfully used to associate *Dicer-2* and *Argonaute 2* (*AGO2*) with the RNAi pathway in WCR [48]. For this purpose, genes encoding proteins active in steps of the endocytic pathway and possibly involved in RNAi response in other insects [32, 33, 39–43] were silenced, and the effect on *laccase2* gene knockdown was analyzed.

Gene knockdown of *Chc*, *Vha16*, *AP50*, *Arf72A*, and *Rab7* was evaluated in WCR adults after the first dsRNA exposure (stage 1) and two days after the second dsRNA exposure (stage 2) (Fig 1). In this experiment, *lacasse2* was used as the reporter gene instead of *V-ATPase A* as reported by Velez et al. [48], since *Chc* and *Vha16* dsRNA generate mortality, which could lead to misinterpretation of RNAi-derived phenotype. Moreover, both the *V-ATPase A* subunit (an ortholog of *Vha68-2*) and *Vha16* encode subunits of the same functional protein complex [53], hence *V-ATPase A* was not used as a reporter. The expression levels of all five target genes were strongly reduced after the first dsRNA exposure and remained suppressed after the second dsRNA exposure to *laccase2* or *GFP* dsRNA (Fig 2). These results suggest that the expression of the endocytic genes was unaffected by the exposure to a second dsRNA and the knockdown did not decrease during the time that the WCR adults were exposed to the second dsRNA.

**Fig 3:**
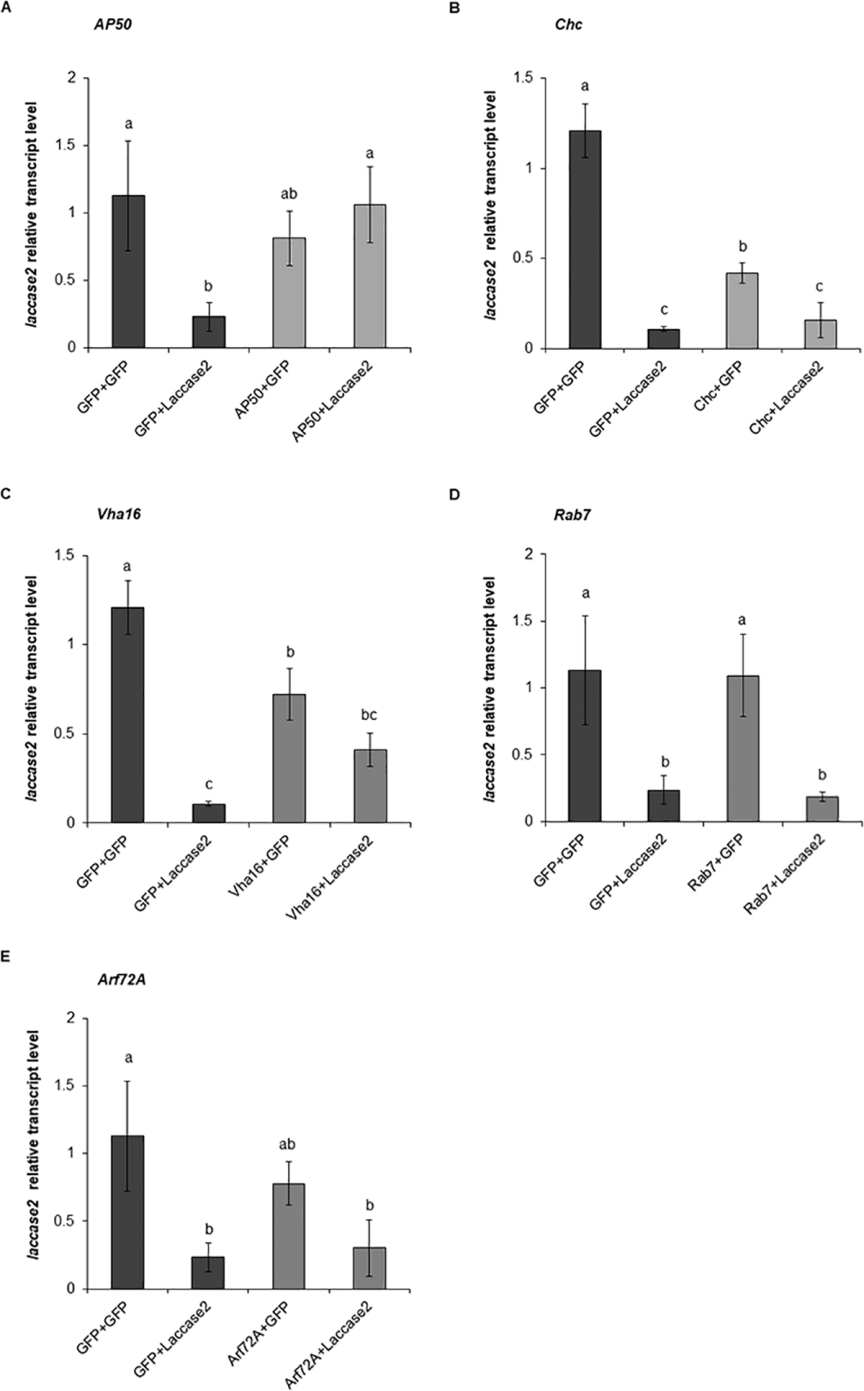
Effect of the knockdown of the endocytosis-related genes on the RNAi response. Effect of the knockdown of the *AP50* (A), *Chc* (B), *Vha16* (C), *Rab7* (D), and *Arf72A* (E) on the relative transcript level of *laccase2* was evaluated by RT-qPCR analysis. Values shown are the means and standard errors (±SE) of three biological replicates each with two technical replicates. Different letters represent significant differences at *p*-value < 0.05.

In WCR adults fed with *GFP* dsRNA and later with *laccase2* dsRNA, the *lacasse2* expression decreased by 79.4%, relative to the control in which WCR adults were treated at both stages with *GFP* dsRNA. In contrast, when WCR adults were exposed to *AP50* dsRNA, followed by *laccase2* dsRNA, the *laccase2* expression was reduced by only 5.9% (Fig 3A). This result strongly suggests that the down-regulation of *AP50* significantly antagonizes the knockdown of *laccase2*, indicating that *AP50* plays an important role in the RNAi response in WCR adults.

We did not observe significant increase in *laccase2* expression when the WCR adults were fed with *Chc* or *Vha16* dsRNAs and subsequently treated with *laccase2* dsRNA compared to beetles fed with GFP dsRNA and later with *laccase2* dsRNA (Fig 3B and 3C). However, it was observed that both *Chc* and *Vha16* dsRNAs caused a reduction in *laccase2* transcript levels, 65.3% and 40.4%, respectively (Fig 3B and 3C). Therefore, it appears that the knockdown of *Chc* and *Vha16* genes also affected the expression of the reporter gene. Similar effects were observed in *Metaseiulus occidentalis* and *Locusta migratoria*, in which *Chc* silencing reduced the expression of the reporter genes, *cathepsin* and *methoprene-tolerant* by 40% and 88%, respectively [54].

Consequently, to prevent confounding results from the *Chc+laccase2* and *Vha16+laccase2* treatments by this non-specific effect, we subsequently used a different approach, as described by Wu and Hoy [54], for the calculation of the reporter gene knockdown in order to evaluate the effect of *Chc* and *Vha16* silencing in *laccase2* expression. In this approach, *Chc+GFP* and *Vha16+GFP* were used as controls to calculate *laccase2* knockdown in *Chc+laccase2* and *Vha16+laccase2* treatments.

The gene silencing of *laccase2* in insects that were first treated with *GFP* dsRNA, followed by *laccase2* dsRNA (*GFP*+*GFP* and *GFP*+*laccase2*) was 91.1% (Fig 3B). Insects treated with *Chc* dsRNA and subsequently with *laccase2* dsRNA (*Chc*+*GFP* x *Chc*+*laccase2*) showed a reduction of *laccase2* expression by 62.5% (Fig 3B), while in beetles treated with *Vha16* dsRNA and later with *laccase2* dsRNA (*Vha16*+*GFP* and *Vha16*+*laccase2*) the *laccase2* expression decreased by 43.0% (Fig 3C). These data indicate that *Chc* and *Vha16* transcript suppression reduced the expression of *laccase2* by 28.6% and 48.1%, respectively (Fig 3B and 3C); reinforcing our hypothesis that clathrin-dependent endocytosis is required for RNAi response in WCR adults. The *Arf72A* and *Rab7* silencing, however, did not result in significant retention of *laccase2* expression (Fig 3D and 3E).

### The effect of *silA* and *silC* genes silencing on RNAi response

**Fig 4:**
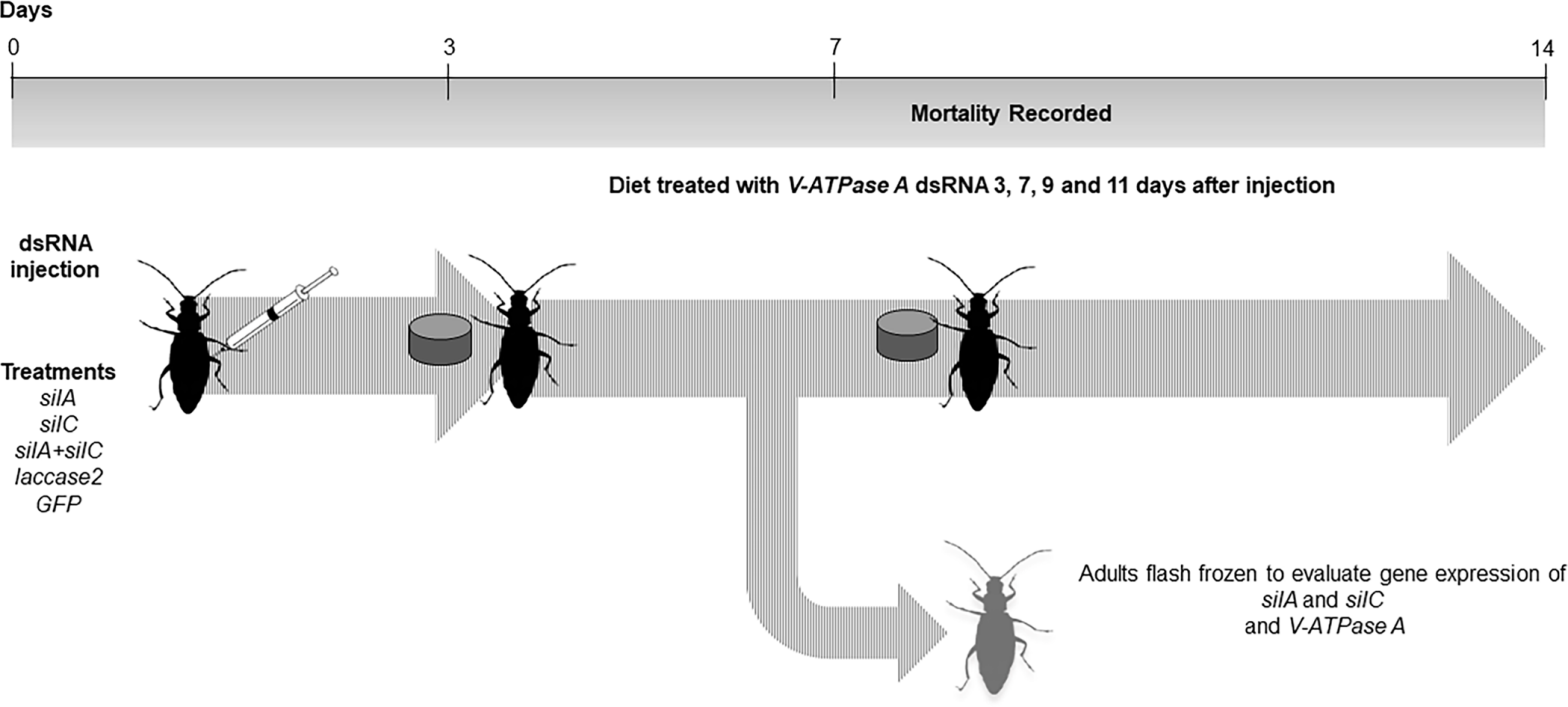
Two-step bioassay to determine the effect of the suppression of *silA* and *silC* genes on the mortality and *V-ATPase A* expression in WCR adults.

To determine if the SIL proteins are involved in the RNAi response in WCR adults, we performed experiments using an “RNAi-of-RNAi” approach described by Velez et al. [48] (Fig 4). In a previous study, feeding of WCR adults on artificial diet treated with dsRNA of the *Vacuolar-ATPase subunit A* (*V-ATPase A*) gene caused high mortality after 14 days of exposure [22]. Therefore, we chose *V-ATPase A* as a reporter gene to investigate the role of the SILA and SILC proteins in the RNAi response in WCR adults (Fig 4). The WCR *V-ATPase A* is most closely related to *Drosophila melanogaster Vha68-2* (CG3762), and the predicted V-type proton ATPase catalytic subunit A (XP_976188.1) of *Tribolium castaneum*. To determine if competition for the RNAi machinery occurred between the dsRNAs from the first and the second exposures, a treatment group in which the WCR adults were first injected with *laccase2* dsRNA, followed by feeding with *V-ATPase A* dsRNA was included. *Lacasse2* is involved in cuticular tanning [49] and is not associated with RNAi response.

**Fig 5:**
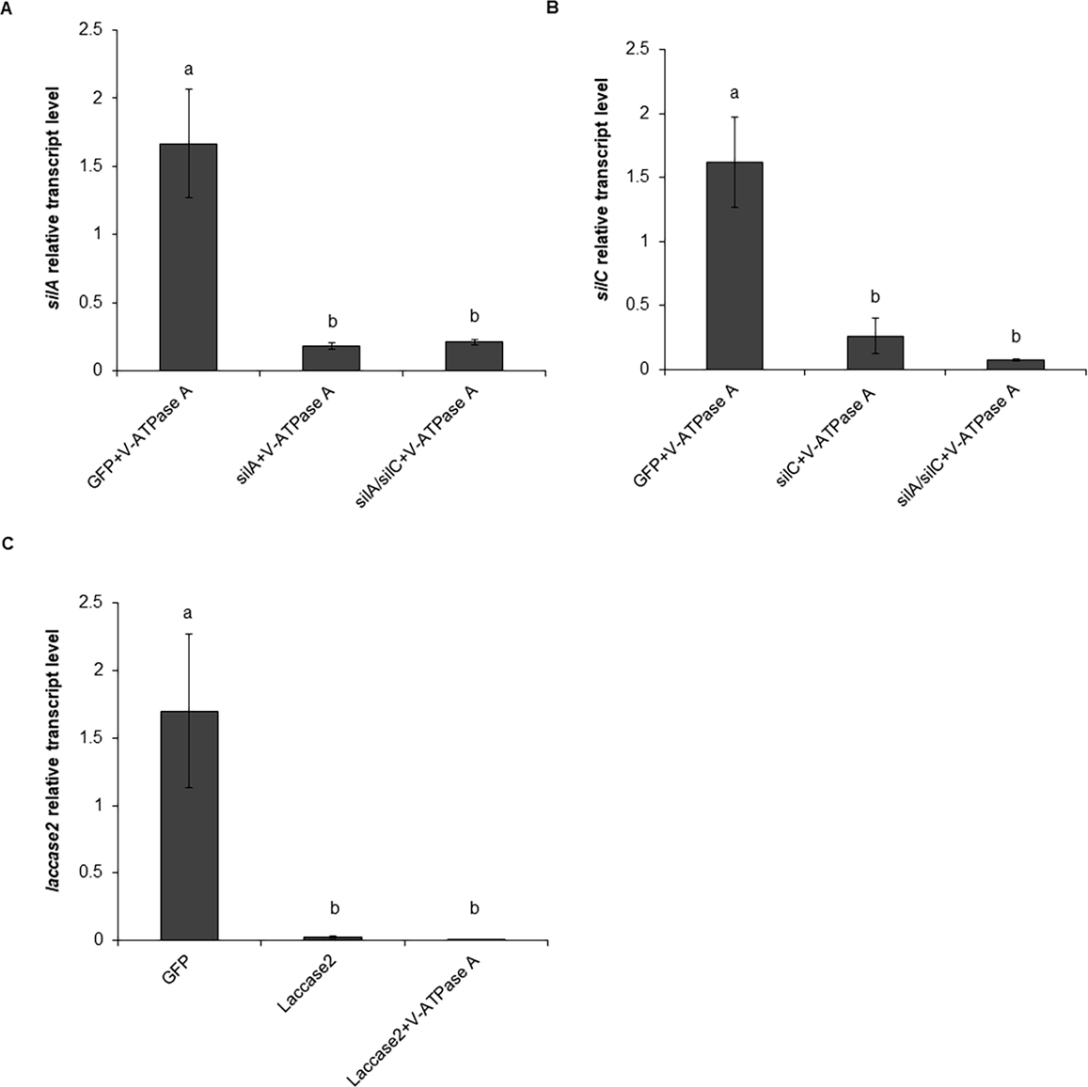
Relative transcript level of *silA*, *silC* and *laccase2* genes after dsRNA exposure. Relative transcript level of *silA* (A), *silC* (B) and *laccase2* (C) evaluated seven days after the injection of 600 ng of the respective dsRNA or a combination of *silA* and *silC* dsRNAs into WCR adults. Values shown are the means and standard errors (±SE) of three biological replicates, each with two technical replicates. Different letters represent significant differences at *p*-value < 0.05.

Seven days after dsRNA injections a robust reduction in expression of the *silA*, *silC*, and *laccase2* genes was observed (Fig 5). Knockdown of *silA* and *silC* genes was achieved by injection of dsRNA rather than feeding, as we have previously observed higher and more consistent rates of knockdown using an injection. In the treatment group *silA+V-ATPase A* and *silA/silC+V-ATPase A*, the expression of *silA* was significantly reduced compared to the control treatment (adults injected with *GFP* dsRNA and subsequently fed with *V-ATPase A* dsRNA) by 89.1% and 87.5%, respectively (Fig 5A). The expression of *silC* in the treatment group *silC+V-ATPase A* and *silA/silC+V-ATPase A* was reduced by 83.9% and 95.5%, respectively, and was significantly different from the control (Fig 5B). Additionally, the reduction of *laccase2* expression was 98.8% and 99.6% when the WCR adults were injected with *laccase2* dsRNA and subsequently fed with untreated diet or *V-ATPase A* dsRNA, respectively (Fig 5C).

**Fig 6:**
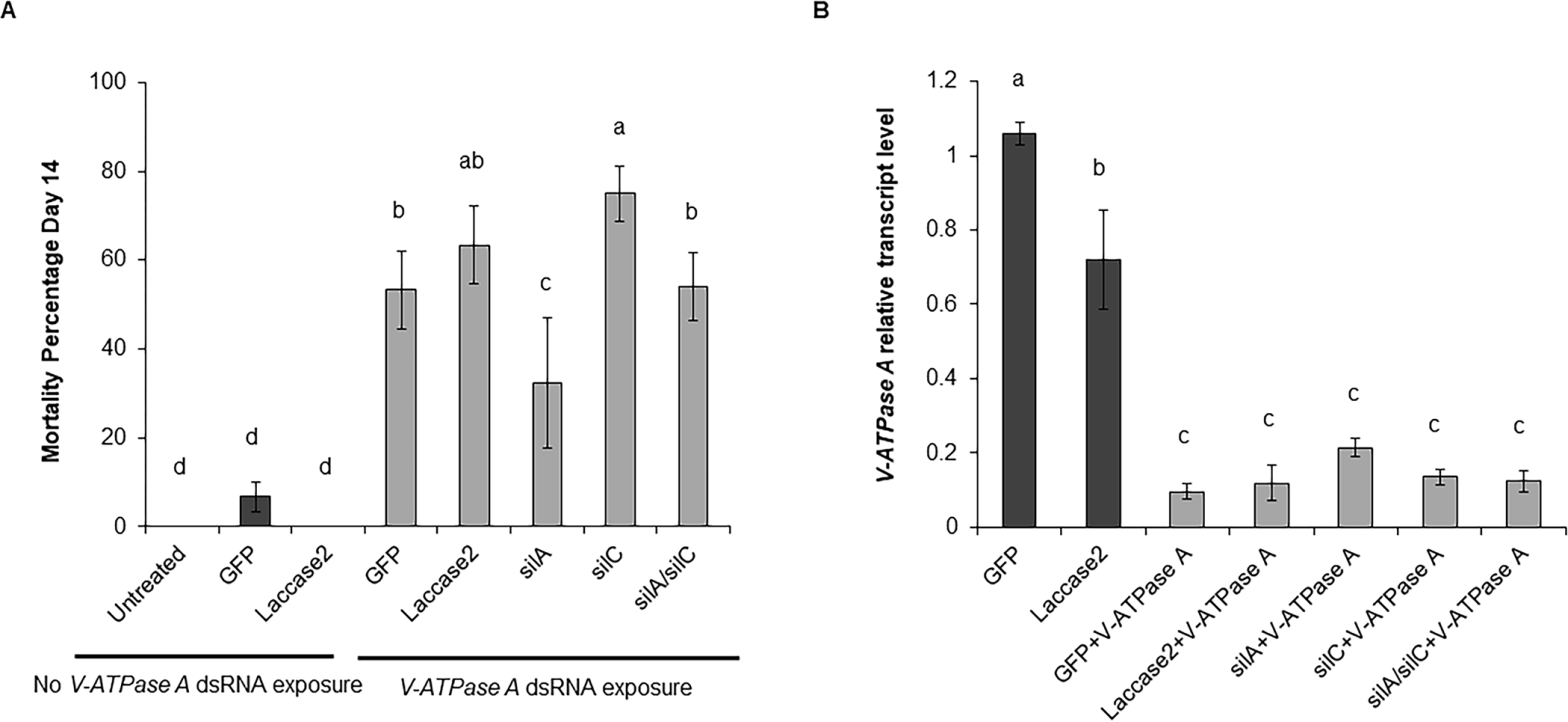
Mortality of WCR adults and relative *V-ATPase A* expression. (A) Mortality of WCR adults from the different treatment groups after 14 days. WCR adults were injected with 600 ng of *GFP*, *laccase2*, *silA*, *silC*, or a combination of *silA* and *silC* dsRNAs and subsequently fed with *V-ATPase A* dsRNA. WCR adults injected with *GFP* and not exposed to *V-ATPase A* dsRNA were used as controls. (B) Relative *V-ATPase A* transcript levels were evaluated by RT-qPCR seven days after exposed to *V-ATPase A* dsRNA. Values shown are the means and standard errors (±SE) of three biological replicates. Different letters represent significant differences at *p*-value < 0.05.

The knockdown of *laccase2* did not affect the knockdown of the *V-ATPase A* gene or the mortality of WCR adults, when compared with control insects injected with *GFP* dsRNA and fed with *V-ATPase A* dsRNA (Fig 6A and 6B). These results indicate that mortality and *V-ATPase A* expression of the treatment groups injected with *sil* dsRNA and fed with *V-ATPase A* dsRNA were not due to potential competition between dsRNAs. Furthermore, the concurrent silencing of *silA* and *silC* genes suggests that oversaturation of the RNAi machinery did not occur when a mixture of *silA* and *silC* dsRNAs was injected, at least at dsRNA concentrations that were evaluated in this assay (Fig 5A and 5B).

The down-regulation of the *silC* gene did not significantly suppress the mortality of the WCR adults, which were injected with *silC* dsRNA or with the mixture of *silA* and *silC* dsRNAs and subsequently fed with *V-ATPase A* dsRNA, compared to the control (Fig 6A). Furthermore, *V-ATPase A* transcript abundance was not significantly influenced by the down-regulation of the *silC*, implying that in WCR adults, SILC protein is not required for effective RNAi response (Fig 6B). When WCR adults were injected with *silA* dsRNA and fed with *V-ATPase A* dsRNA, no significant increase in *V-ATPase A* transcript abundance was observed. However, the mortality of the WCR adults was significantly reduced (Fig 6A), suggesting that the SILA protein may play a role in the RNAi response.

## Discussion

Most studies aimed at understanding the mechanisms of cellular dsRNA uptake and the systemic spread of RNAi effect in insects have focused on SIL proteins and the endocytic pathway as key participants [31–33, 39–43]. Since the level of environmental RNAi response varies greatly from one insect to another, the genes involved in this response may also vary. To elucidate which gene products are involved in dsRNA uptake in WCR, the present work investigated the impact of knocking down several components of clathrin-dependent endocytosis and homologs of SID-1 on the RNAi response in WCR beetles. The genes evaluated in this study included WCR endocytosis-related targets *Chc*, *Vha16*, *AP50*, *Arf72A*, *Rab7* and SID-like genes *SilA* and *SilC*.

Role of the endocytic pathway in dsRNA uptake has been demonstrated in *D*. *melanogaster* S2 cells, and confirmed in *S*. *gregaria*, *L*. *decemlineata*, *T*. *castaneum* and *B*. *dorsalis*, suggesting that this mechanism of dsRNA internalization might be widespread among insects [32, 33, 39–42]. A study by Ulvila et al. [33] confirmed that *Chc* was necessary for dsRNA uptake in *Drosophila* S2 cells, by bypassing this pathway via transfection. Further, studies in S2 cells [32] and *T*. *castaneum* [41] have confirmed the involvement of clathrin-mediated endocytosis in RNAi response by pharmacological block via bafilomycin-A1 and bafilomycin-A1 or chlorpromazine, respectively. Xiao et al. [41] showed that both bafilomycin-A1 and chlorpromazine can block the uptake of fluorescently-labeled dsRNA by larval midgut cells. While by their nature, the RNAi-of-RNAi experiments performed in the current study cannot identify which part of RNAi process is perturbed, by extension, the genes identified in our study as key participants in RNAi response are likely to be involved in dsRNA uptake.

In the present study, knockdown of WCR endocytic genes, *AP50*, *Chc*, and *Vha16* reduced the subsequent knockdown of the reporter gene *laccase2* (Fig 3A–3C), confirming that the endocytic pathway participates in the RNAi response. Results from this study support the observations in coleopteran insects *L*. *decemlineata* and *T*. *castaneum*, where silencing of endocytosis-related genes *Chc* and *Vha16*, reduced the subsequent RNAi response [41–43]. Anchoring on *AP50*, *Chc*, and *Vha16* results, we identified clathrin-mediated endocytosis as being essential for a robust RNAi response in WCR.

**Fig 7:**
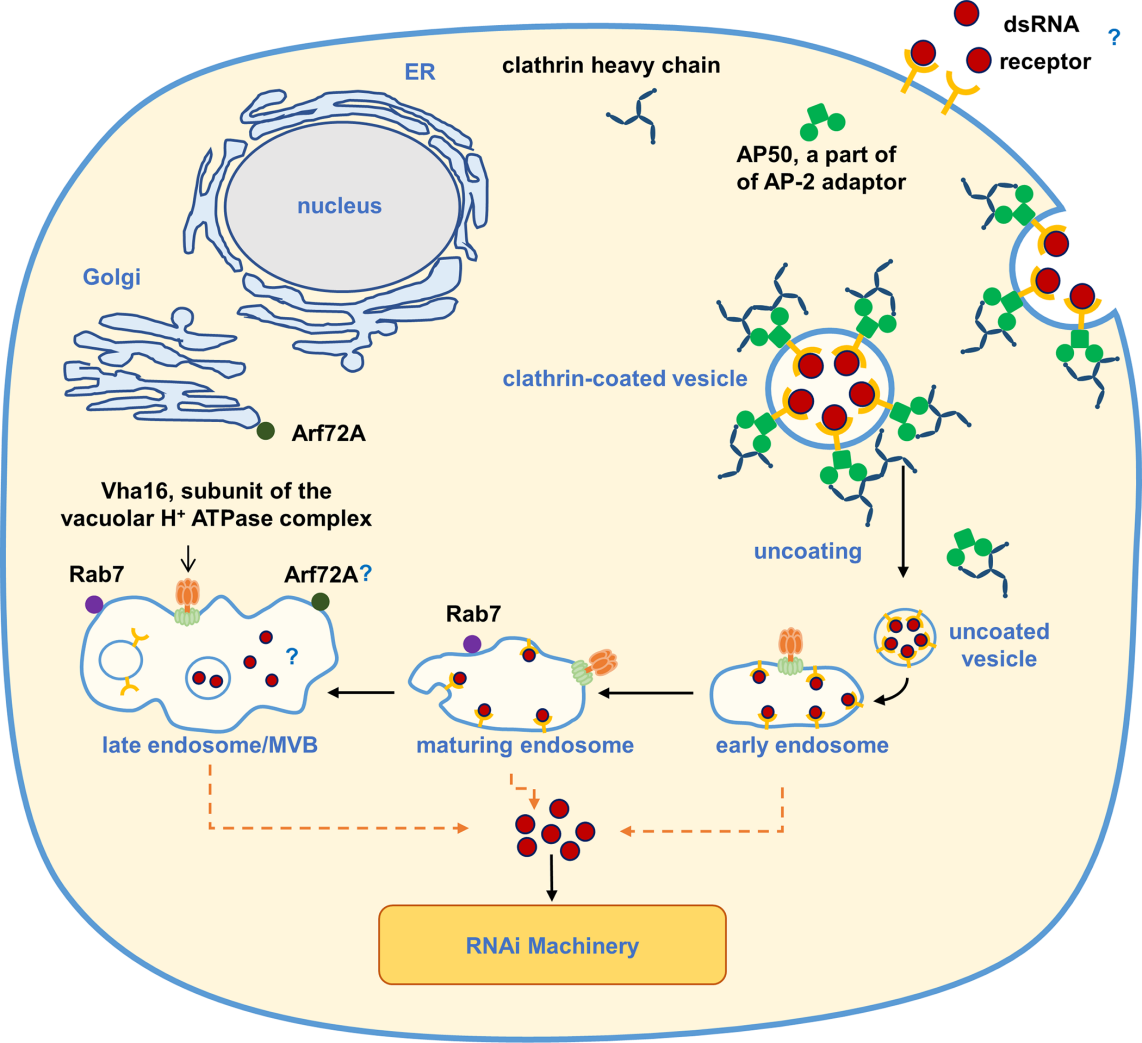
Known components of clathrin-mediated endocytosis involved in the RNAi response in WCR.

In contrast to the robust effects of *AP50*, *Chc*, and *Vha16* on RNAi response in WCR, knockdown of *Arf72A* and *Rab7* did not result in significant increase of *laccase2* expression (Fig 3D and 3E). Although we observed no effect for *Rab7* and *Arf72A*, partial block of the RNAi response was observed in *L*. *decemlineata* (Lepd-SL1) cells and *T*. *castaneum* when these genes were silenced [41, 43]. Unlike Chc or Vha16, which may be necessary throughout the endocytic cycle, Rab7 is associated with late endosomes/multivesicular bodies. Arf72A/Arl1 is known to associate primarily with the Golgi network [35, 55] and binds AP1 clathrin adaptor [37] (Fig 7). To become available to the RNAi machinery in the cytosol, the dsRNA needs to escape from the early to late endosomes before they fuse with lysosomal compartments [56]. It is hence possible that sufficient *laccase2* dsRNA escaped in the earlier steps of the endocytosis, thus the knockdown of *Arf72A* and *Rab7* genes did not significantly affect the expression of *laccase2*. These hypotheses are further supported by the fact that in coleopteran insects dsRNA escape from the endosomal compartments appears to be an efficient process leading to a robust RNAi response [57].

The RNAi-of-RNAi approach used in this study implicated Clathrin heavy chain (Chc), Clathrin adaptor protein AP50 (AP50), and Vacuolar H^+^ ATPase 16 kDa subunit (Vha16) in the RNAi response of WCR. Rab7 is necessary for clathrin-mediated endocytosis, it associates with late endosomes/multivesicular bodies and may function downstream of the dsRNA release into cytoplasm. Arl1/Arf72A associates with trans-Golgi.

In the case of Arf72A, it is possible that its effect on the uptake of dsRNA is indirect, since blocking Arl1/Arf72A causes the dispersion of AP1, which affects secretory granule biogenesis and clathrin exchange [37]. Similarly, depletion of Arl1 protein (Arl1p) in *Saccharomyces cerevisiae* leads to both decreased protein uptake and secretion since the regulation of membrane traffic is disturbed [58]. Therefore, the block of Arf72A seems to perturb important processes involved in the clathrin pathway, which could interfere in the dsRNA uptake or transport.

The insect studies of SID-like genes have been driven largely by the knowledge from systemic RNAi deficiencies (SID) identified in *C*. *elegans* mutagenesis screens. The transmembrane protein systemic RNA interference deficiency-1 (SID-1) is necessary for the uptake of dsRNA in *C*. *elegans*, enabling systemic spread of the RNAi effect [24]. The presence of *C*. *elegans sid-1* homologues has been confirmed in multiple insects including *Schistocerca americana*, *Spodoptera exigua*, *Spodoptera litura*, *Spodoptera frugiperda*, *Aphis glycines*, *Aphis gossypii*, *Anthonomus grandis*, *Nilaparva lugens*, *Apis mellifera*, *L*. *decemlineata*, and *D*. *v*. *virgifera* larvae [31, 42, 59–67]. However, as observed in our study, the presence of *sil* genes in insects does not assure the participation of SIL proteins in RNAi response. In our study, only *silA* significantly suppressed the *V-ATPase A* knockdown phenotype in WCR adults (Fig 6A). However, this data was not reflected by a commensurate reduction in *V-ATPase A* transcript levels (Fig 6B). We did not observe an impact of *silC* on the RNAi response at either transcript or phenotypic levels. Nevertheless, since the nature of RNAi-of-RNAi experiments does not assure full knockdown of the gene targets, it is possible that some SILA or SILC protein remained. Additionally, the half-life of the SIL proteins may allow them to remain functional while their transcripts are largely depleted. Therefore, the participation of *D*. *v*. *virgifera* SILA or SILC proteins in dsRNA response cannot be ruled out.

The participation of the *sil* genes in the RNAi response has been suggested in *N*. *lugens*, *L*. *decemlineata* and *D*. *v*. *virgifera* larvae [31, 42, 66]. In *L*. *decemlineata*, it has been reported that *silA* and *silC* are necessary for an effective RNAi response [42, 43]. Further, the silencing of *silA* promoted stronger suppression of reporter gene knockdown compared to the silencing of *silC* [42]. A study performed with WCR larvae demonstrated through an “RNAi-of-RNAi” approach that the silencing of *silA* and *silC* genes suppressed the RNAi phenotype of the *ebony* gene, suggesting the involvement of both *sil* genes in dsRNA response [31]. However, the phenotype resulting from *ebony* RNAi was not pronounced [31], suggesting that these proteins probably are not the unique mechanism associated with the uptake of dsRNA in WCR.

Reports for other insects including *L*. *migratoria*, *S*. *gregaria*, *Plutella xylostella* and *T*. *castaneum* indicate that the *sil* genes are not involved in systemic RNAi [30, 39, 68, 69]. Detailed sequence analysis of the SIL proteins has revealed that in some insects the N-terminal extracellular domain shares more identity with the TAG-130/CHUP-1 protein of *C*. *elegans*, involved with cholesterol internalization, than with the SID-1 protein [30, 68]. We also noted that both WCR SILA and SILC are more similar to the *C*. *elegans* cholesterol uptake protein CHUP-1(S1 Fig). Thus, it is possible that the WCR SILA and SILC proteins perform functions associated with cholesterol uptake rather than dsRNA transport [70], and any CHUP-1-associated phenotypes may occur indirectly, through interference with cholesterol endocytic pathway [71] with subsequent impact on membrane transport.

In summary, the comparison of clathrin-mediated endocytosis and *sil* genes in the RNAi response of WCR suggests that clathrin-dependent endocytosis is more likely to be the primary mechanism for the import of dsRNA in this insect. Further experiments using either WCR biological stages or a coleopteran cell culture similar to those performed by Yoon et al. [43] and Yoon et al. [72] focused on monitoring dsRNA uptake by cells and its movement will help shed light on dsRNA uptake and spread in WCR. Overall, our research provides a starting point for future studies, which surely will have important implications for the development, efficacy, and improvement of RNAi-based management strategies directed to control WCR populations.

## Supporting information

S1 Table Primer sequences used for dsRNA synthesis. (DOCX)

S2 Table Primer sequences and parameters used for qRT-PCR analysis. (DOCX)

S3 Table Genes found in WCR transcriptome and their percentage of amino acid sequence identity with other insect species. (DOCX)

S1 Fig Sequence alignments and phylogenetic relationships of SIL proteins from WCR. (DOCX)
