# Supplementary material for: Clathrin-dependent endocytosis is associated with RNAi response in the western corn rootworm, Diabrotica virgifera virgifera LeConte
Source: PLoS One. 2018 Aug 9;13(8):e0201849. doi: 10.1371/journal.pone.0201849 (PMC6084943; doi:10.1371/journal.pone.0201849)
Supplement: S1 Table — (DOCX) [file pone.0201849.s001.docx]

**S1 Table.** Primer sequences used for dsRNA synthesis.

| **Primer** | **Sequence (5’- 3’) for dsRNA synthesis** | **Product size (bp)** |
| --- | --- | --- |
| ds*GFP*-F | TAATACGACTCACTATAGGGGGTGATGCTACATACGGAAAG | 370 |
| ds*GFP*-R | TAATACGACTCACTATAGGGTTGTTTGTCTGCCGTGAT |  |
| ds*laccase2*-F | TAATACGACTCACTATAGGGATGTGCAAGAGCTTGTAGGG | 183 |
| ds*laccase2*-R | TAATACGACTCACTATAGGGATGCGATTGGCTGTTAGAAG |  |
| ds*V-ATPase A* -F | TAATACGACTCACTATAGGGTATTGTACAGGTG | 258 |
| ds*V-ATPase A* -R | TAATACGACTCACTATAGGGCAATTTCCAAG |  |
| ds*silA*_F | TAATACGACTCACTATAGGGCAGAACCGTCATCCAGATA | 382 |
| ds*silA*-R | TAATACGACTCACTATAGGGCCATCAATAACGCTAACAAGA |  |
| ds*silC*-F | TAATACGACTCACTATAGGGGTTTTGAGCGCAAGTTACCA | 374 |
| ds*silC*-R | TAATACGACTCACTATAGGGTGGCACGGTTTTATGACATT |  |
| ds*Chc*-F | TTAATACGACTCACTATAGGGAGA GGAAGATTGGCTGATTTGGA | 291 |
| ds*Chc*-R | TTAATACGACTCACTATAGGGAGA CACGATGTGCATACCACACA |  |
| ds*Vha16*-F | TTAATACGACTCACTATAGGGAGA TCATGAGGCCGGAACTTATC | 327 |
| ds*Vha16*-R | TTAATACGACTCACTATAGGGAGA AGAGGTAAATGGCGACGATG |  |
| ds*AP50*-F | TTAATACGACTCACTATAGGGAGA CTCCCGATGGAGAATTTGAA | 277 |
| ds*AP50*-R | TTAATACGACTCACTATAGGGAGA GCCATTCGCTTTATTTTCCA |  |
| ds*Arf72A*-F | TTAATACGACTCACTATAGGGAGA TTGGGATTAGACGGTGCAG | 200 |
| ds*Arf72A*-R | TTAATACGACTCACTATAGGGAGAATGATTGCATCTGTATTGCTGTAG |  |
| ds*Rab7*-F | TTAATACGACTCACTATAGGGAGA AATTCCTCATCCAAGCATCG | 299 |
| ds*Rab7*-R | TTAATACGACTCACTATAGGGAGA ATCACCGTTGGTGTTGGTTT |  |
| F: Forward primer; R: Reverse primer; Underlined sequence: T7 promoter | |  |
