# Supplementary material for: Clathrin-dependent endocytosis is associated with RNAi response in the western corn rootworm, Diabrotica virgifera virgifera LeConte
Source: PLoS One. 2018 Aug 9;13(8):e0201849. doi: 10.1371/journal.pone.0201849 (PMC6084943; doi:10.1371/journal.pone.0201849)
Supplement: S2 Table — (DOCX) [file pone.0201849.s002.docx]

**S2 Table.** Primer sequences and parameters used for qRT-PCR analysis.

| **Primer** | **Sequence (5’- 3’) for qRT-PCR** | **Product size (bp)** | **Slope** | **R^2^** | **% Efficiency** |
| --- | --- | --- | --- | --- | --- |
| qPCR*laccase2*-F | GAGCAGCTTGCCAAGTATGT | 106 | -3.169 | 0.998 | 106.8 |
| qPCR*laccase2*-R | ATGCGATTGGCTGTTAGAAG |  |  |  |  |
| qPCR*V-ATPase*-A-F | GGAAGAAGATGATCTAGCCGAAATT | 67 | -3.361 | 0.993 | 98.4 |
| qPCR*V-ATPase A* -R | TTGTCCGTTTCTGCCAGAGA |  |  |  |  |
| qPCR*silA*-F | ACGCACTTAAACCTATACGGAAA | 165 | -3.29 | 0.99 | 101.3 |
| qPCR*silA*-R | CACAATGAATGACGCTGTTACC |  |  |  |  |
| qPCR*silC*-F | GAACTTTCGCACAAAGACGA | 116 | -3.457 | 0.996 | 94.6 |
| qPCR*silC*-R | TAACTTGCGCTCAAAACACC |  |  |  |  |
| qPCR*Chc*-F | GGCCAGAGAGAGCTACATCG | 196 | -3.437 | 0.998 | 95.4 |
| qPCR*Chc*-R | AGCTAGACGAGCGAAGTTGG |  |  |  |  |
| qPCR*Vha16*-F | TCCTCATTTTCGCCGAAGTA | 124 | -3.251 | 0.999 | 103 |
| qPCR*Vha16*-R | GCAACTTTTGGTACGCTGTG |  |  |  |  |
| qPCR*AP50*-F | ATCGGAGAATGCCATTGTGT | 112 | -3.482 | 0.998 | 93.7 |
| qPCR*AP50*-R | GCGAGTCCATTTCTTTTTGG |  |  |  |  |
| qPCR*Arf72A*-F | ATTTAGGGGGCCAGACAAGT | 147 | -3.493 | 0.99 | 93.3 |
| qPCR*Arf72A*-R | GCTCTTCTTCCCTTAACATTGG |  |  |  |  |
| qPCR*Rab7*-F | CCTCATTCAAATCCCTGGAC | 109 | -3.339 | 0.993 | 99.3 |
| qPCR*Rab7*-R | TCGAGGTCGACTTTGTTTCC |  |  |  |  |
| qPCR*β-actin*-F | TCCAGGCTGTACTCTCCTTG | 134 | -3.419 | 0.999 | 96.1 |
| qPCR*β-actin*-R | CAAGTCCAAACGAAGGATTG |  |  |  |  |

F: Forward primer; R: Reverse primer

R^2^: Correlation coefficient, Eff. (%): percent primer efficiency
