# Supplementary material for: Clathrin-dependent endocytosis is associated with RNAi response in the western corn rootworm, Diabrotica virgifera virgifera LeConte
Source: PLoS One. 2018 Aug 9;13(8):e0201849. doi: 10.1371/journal.pone.0201849 (PMC6084943; doi:10.1371/journal.pone.0201849)
Supplement: S3 Table — (DOCX) [file pone.0201849.s003.docx]

**S3 Table.** Genes found in WCR transcriptome and their percentage of amino acid sequence identity with other insect species.

| **Genes Identified in**  **WCR** | **Species** | **GenBank Accession Number** | **Percentage Identity to WCR** |  |
| --- | --- | --- | --- | --- |
|  |  |  |  |  |
| *Chc* (KX965603) | *Tribolium castaneum* | NP_001280512.1 | 96 |  |
|  | *Nicrophorus vespilloides* | XP_017771564.1 | 93 |  |
|  | *Locusta migratoria* | AHC70342.1 | 89 |  |
|  | *Zootermopsis nevadensis* | KDR22435.1 | 89 |  |
|  |  |  |  |  |
| *Vha16* (KX965604) | *Tribolium castaneum* | XP_967959.1 | 90 |  |
|  | *Eufriesea mexicana* | XP_017766082.1 | 89 |  |
|  | *Musca domestica* | XP_005184512.1 | 88 |  |
|  | *Ceratitis capitata* | XP_004534551.1 | 88 |  |
|  |  |  |  |  |
| *AP50* (KX965605) | *Tribolium castaneum* | NP_001280510.1 | 99 |  |
|  | *Bombyx mori* | XP_004925170.2 | 98 |  |
|  | *Plutella xylostella* | XP_011562722.1 | 97 |  |
|  | *Nicrophorus vespilloides* | XP_017771815.1 | 97 |  |
|  |  |  |  |  |
| *Arf72A* (KX965607) | *Tribolium castaneum* | XP_973025.1 | 94 |  |
|  | *Culex quinquefasciatus* | XP_001843016.1 | 90 |  |
|  | *Anopheles darlingi* | ETN61395.1 | 89 |  |
|  | *Apis mellifera* | XP_001120141.1 | 88 |  |
|  |  |  |  |  |
| *Rab7* (KX965606) | *Tribolium castaneum* | NP_001284604.1 | 92 |  |
|  | *Diachasma alloeum* | XP_015108335.1 | 86 |  |
|  | *Solenopsis invicta* | XP_011162103.1 | 85 |  |
|  | *Harpegnathos saltator* | XP_011141319.1 | 85 |  |
|  |  |  |  |  |
| *silA* (KX965608) | *Leptinotarsa decemlineata* | ALG36906.1 | 67 |  |
|  | *Tribolium castaneum* | NP_001099012.1 | 61 |  |
|  | *Nicrophorus vespilloides* | XP_017769437.1 | 49 |  |
|  | *Zootermopsis nevadensis* | KDR19041.1 | 46 |  |
|  |  |  |  |  |
| *silC* (KX965609) | *Leptinotarsa decemlineata* | ALG36907.1 | 80 |  |
|  | *Tribolium castaneum* | NP_001099128.1 | 73 |  |
|  | *Nicrophorus vespilloides* | XP_017777403.1 | 62 |  |
|  | *Cephus cinctus* | XP_015588840.1 | 58 |  |
