# Supplementary material for: Clathrin-dependent endocytosis is associated with RNAi response in the western corn rootworm, Diabrotica virgifera virgifera LeConte
Source: PLoS One. 2018 Aug 9;13(8):e0201849. doi: 10.1371/journal.pone.0201849 (PMC6084943; doi:10.1371/journal.pone.0201849)
Supplement: S1 Fig — (DOCX) [file pone.0201849.s004.docx]

**S1 Fig**

**A**


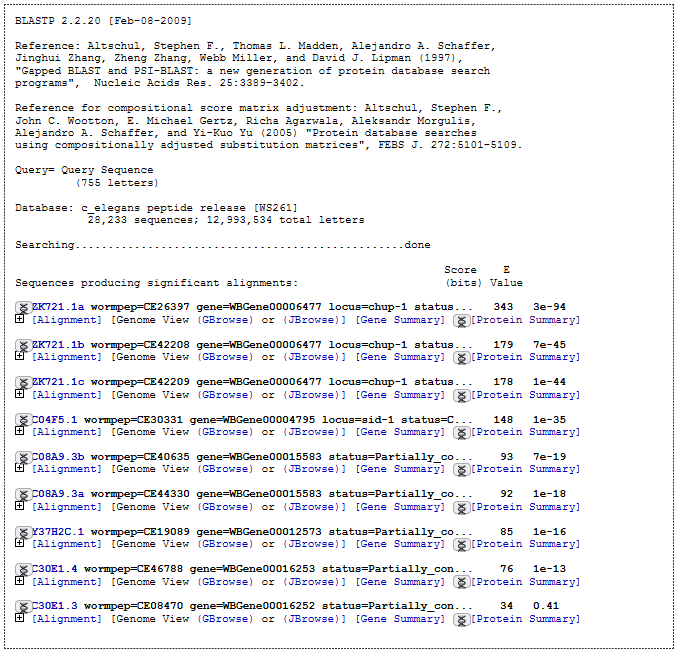


**B**


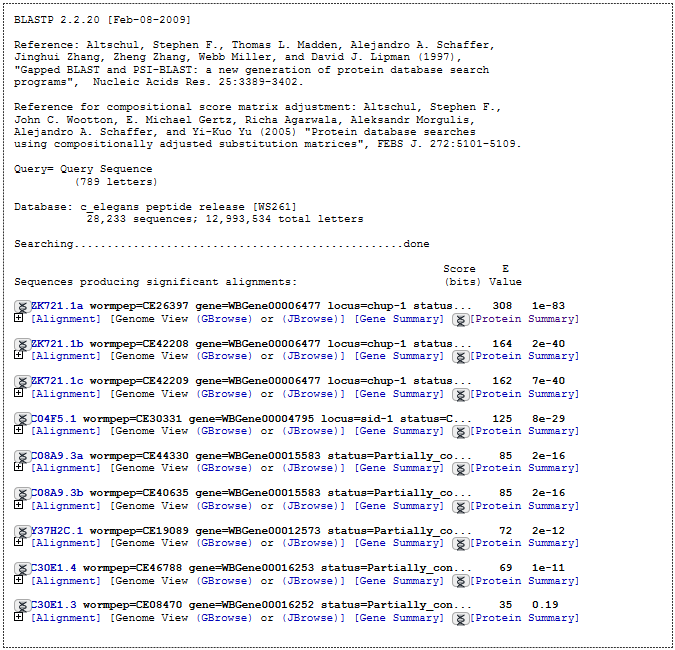


**C**
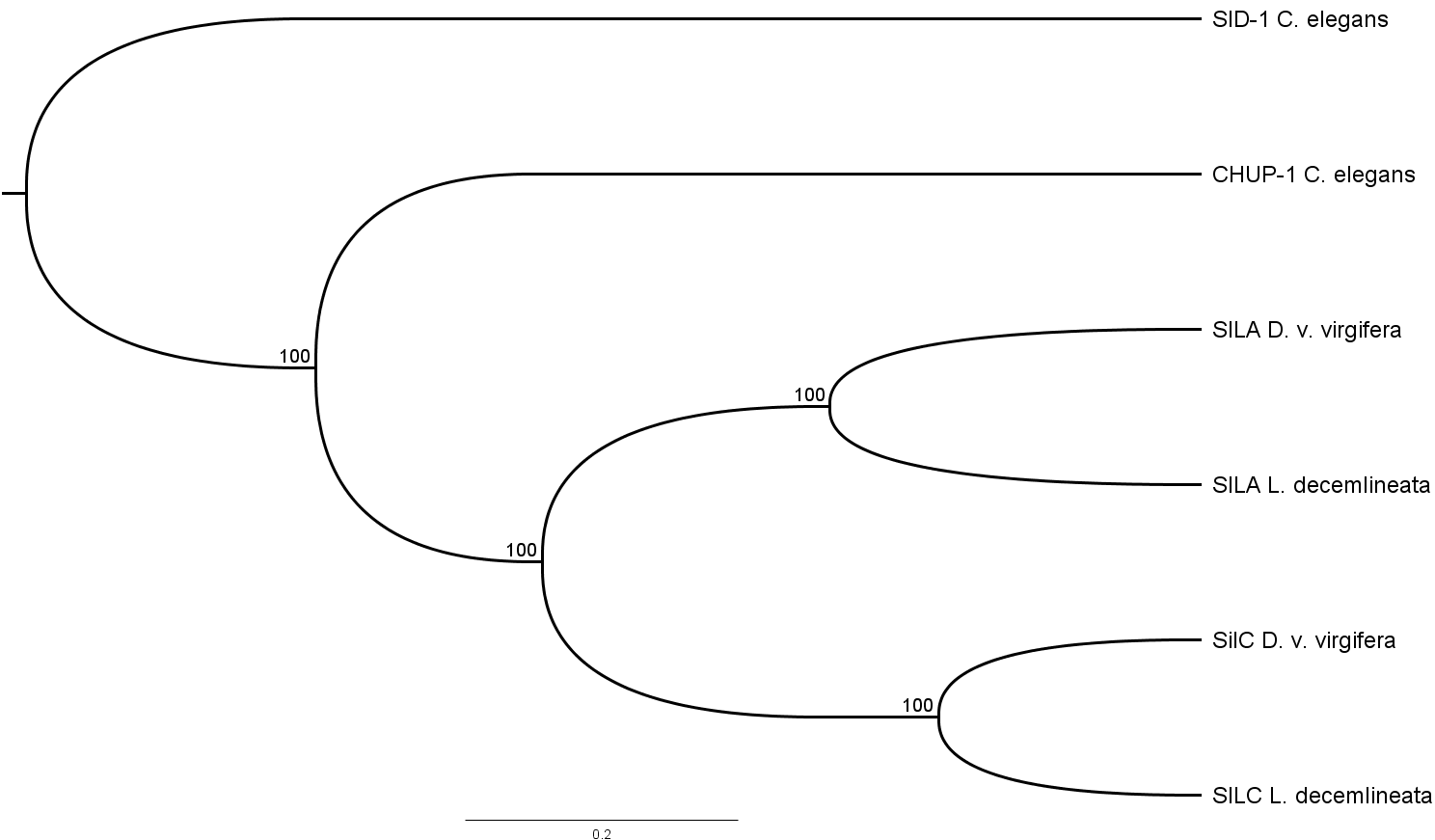


**S1 Fig. Sequence alignments and phylogenetic relationships of SIL proteins from WCR**

**(A)** WormBase <http://www.wormbase.org/> BLAST (blastp) results for putative WCR SILA protein: MKNLQWILLAVCFGLINCKSTNNLTIEQQLLTNGSPITNQLNANKQIILLYRNFTSINPYRIITSSENAKKEFPVLIVAEQRTQVTAWSIPMLIESTKPGVEYSYNMSSKTLCHDYMNIIILSPGIMIVRPLYFQQNFIVALSTASTTDVNVTVELKEEPDFYLIQDKPYNMTVGPSEPRYAYFRFEKDTADTVVIEVDSEDELCLTISVQDSKCPVFDNNKDVKYEGIHQTINTKGAITLLKRSYKDGFFLVFVAKPDTYDCSQESSNLPRLTRTVSIEQMVRTHVNFIIRNSITANDYTIAVGATFLILIGSGILVTVAALIFHRYGTIARNKYEDVIVSDYFEALSEEQISSLLRKLDLHVSELSRHPNRIKKRSYNYLYHTLSIAIFYSIPVVQLVFTYQRIVNRTGDEDMCYYNFLCAHPAFRFSDFNHIFSNIGYVIFGIIFICVVADRHRIIKLRKDKGIPVHYGIFHAMGVALIIEGLLSACYHICPSQSNYQFDTSFMYIMAVLCMVKLYQNRHPDINATAYTTFTVLGCAIFMAMIGILNGNLAIWIIFVVCYTLLCLFLSLKIYFLNYVVDGLNQFRSSVTKKGFTQHALKPIRKARFIILLIANIANYSMLICGLLLYSDNVTDFGTFLLALLMGNSVIHCVFYTCMKLISKEKICYEAILYGVLAIVCWGSSSVFFLDAATLWTVTPAESRQWNQECIVFKFFDKHDIWHLLSAPALYFTFMYLMSLDDDILDVEQRDLQVF

The results show top match as WBGene00006477, chup-1.

**(B)** WormBase <http://www.wormbase.org/> BLAST (blastp) results for putative WCR SILC protein:

MHRLLLFSVLILLTCWSVNAQNKYTPIYKNFTYGSNNTMNANQYLEYILEFSKDYDDPEIYLPPRIWIQSDADVTAPLMIVARQKKEMISWQLPLEIEGTNGETNENYTSRTMCHDIIKQYRFAGSKQKLKQENVIVSVTTSSVTNVSFTIRVDNQKDFTLELNKEIEFDITPSGPRYFFYNFTSNDTLLSKGDSNYETVILEVTSEDDICMTVSIQNISCPVFDTNQDVTFRGFYETVNRKGGMNIPKYKFPHGFYVVFVAKPDDYACDKGSGNLDAVTNPDRLKSIKLVIKPSITYSDYVKAVLFTLGSVGAFYIIFGLPYFIYSVKKSLPREMAYVDGNFPTTPSAKMTSVQRTVSAVSGPSVDMADFDTLAEVDTDRDLRLGRGEPYLVDLARKHPKELTRKSYLYLYNVVTVAIFYALPVIQLVITYQRVLNETGQQDLCYYNFLCAHPLGVLGDFNHVFSNIGYVLLGVLFLIITYLRELSHKDDDFDRQFGIPQHYGLFYAMGVALIMEGVLSASYHVCPNFLNFQFDSSFMYVMAVLVMVKLYQNRHPDINANAYTTFGVLAVAVVLAMIGLLEGNVYFWTIFVIMHILMCFYLSVKVYYMGCWSVRDISMQKFRQVWIYDFWSGPVNVIKPCHKARFVLLFLGNLCNWGLAIFAIYKLPKNFPVFLLAIFMANTLLYFVFYIVMKYINKEHVRILTWIFLFMSTLCAVSAMWFFLHKAISWKKTAAQSRQFNVECKLFHFYDSHDIWHFLSATGMFFTFMVLLTLDDDLSHTHHSQIPVF

The results show top match as WBGene00006477, chup-1.

**(C)** Phylogenetic tree representation of *D. v. virgifera* and *L. decemlineata* putative SILA and SILC proteins and *C. elegans* SID-1 and CHUP-1. The alignment was performed in Geneious (Geneious Alignment) using global alignment with free end gaps and Blosum62 cost matrix. The consensus tree was assembled using Geneious Tree Builder Jukes-Cantor genetic distance model and UPGMA tree building method. Bootstrap values were generated using 100 replicates.
